# Supplementary material for: Evaluating and Improving Neonatal Gentamicin Pharmacokinetic Models Using Aggregated Routine Clinical Care Data
Source: Pharmaceutics. 2022 Sep 30;14(10):2089. doi: 10.3390/pharmaceutics14102089 (PMC9609639; doi:10.3390/pharmaceutics14102089)
Supplement: Supplementary file 1 [file pharmaceutics-14-02089-s001.zip › pharmaceutics-1854755-supplementary.pdf]

Supplemental for: Evaluating and improving neonatal gentamicin pharmacokinetic models using aggregated routine clinical care data

Dominic M.H. Tong, Jasmine H. Hughes, and Ron J. Keizer

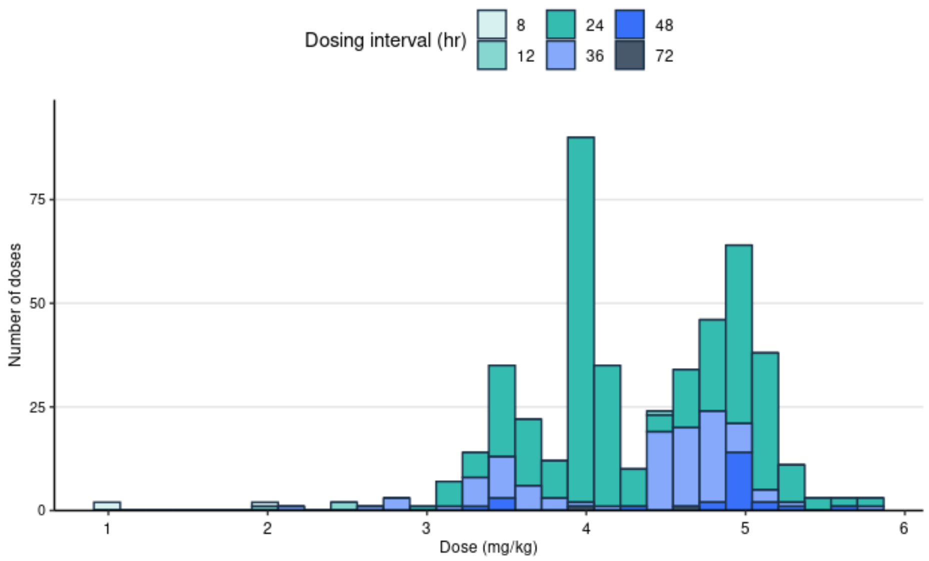

Supplemental Figure S1: Initial gentamicin dosing in mg/kg by estimated dosing interval, which is the actual dosing interval rounded to the nearest values shown.

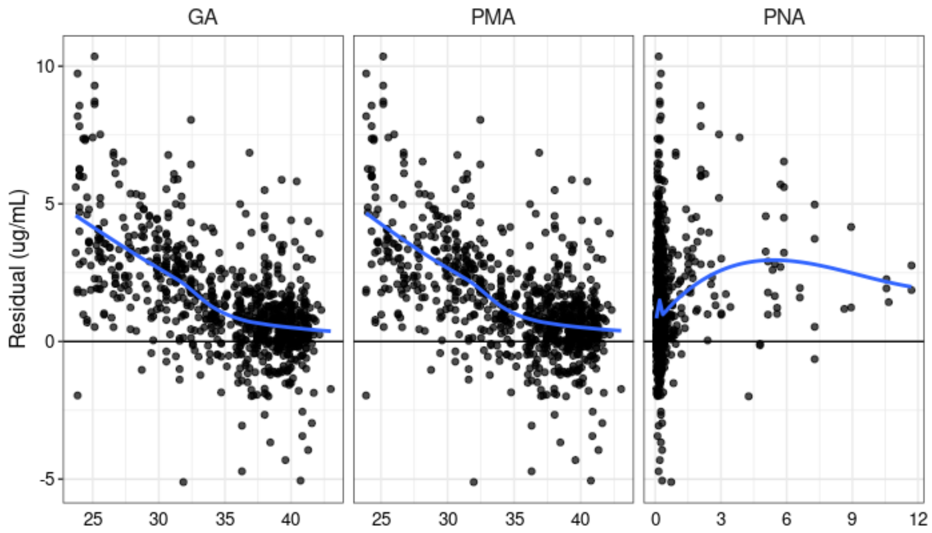

Supplemental Figure S2: Residual (predicted value minus observed value) by gestational age (GA; weeks), postmenstrual age (PMA, sum of gestational age and postnatal age; weeks), and postnatal age (PNA; weeks) using the De Cock model. Blue line represents the locally estimated scatterplot smoothing (LOESS) curve.

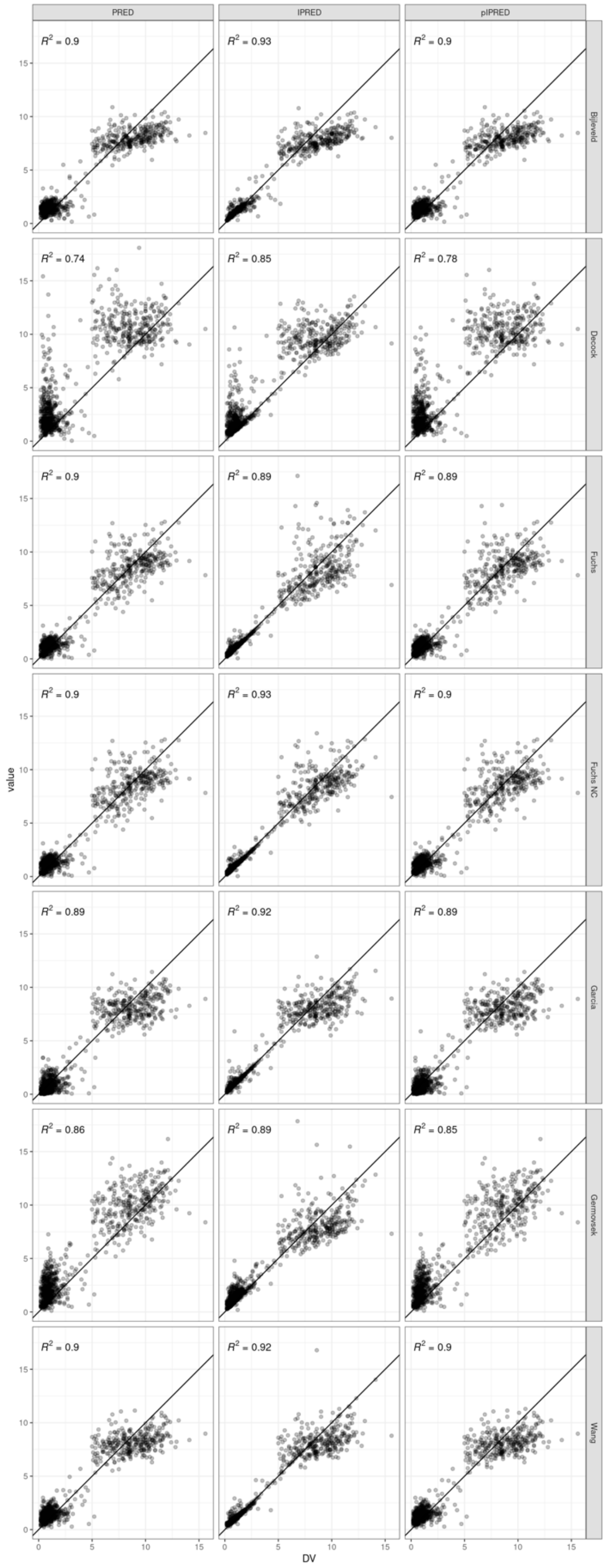

Supplemental Figure S3. Individual prediction (IPRED), population prediction (PRED), and iterative prediction (pPRED) for published gentamicin population pharmacokinetic models on all available data.

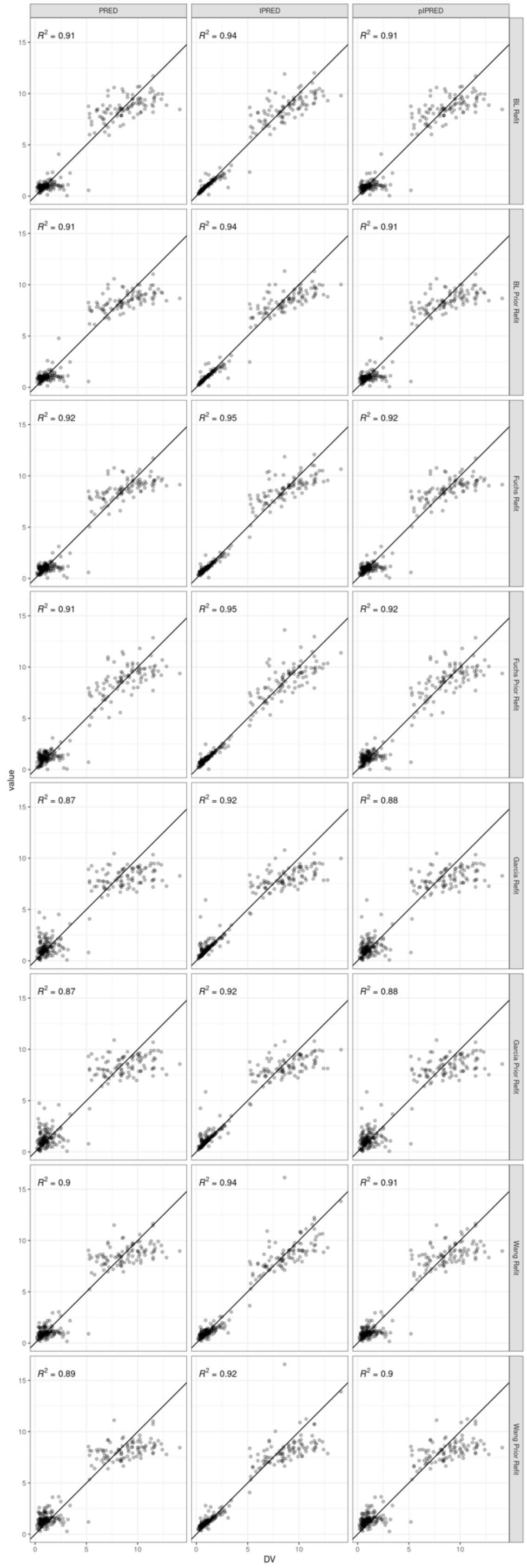

Supplemental Figure S4. Individual prediction (IPRED), population prediction (PRED), and iterative prediction (pPRED) for continuous learning models on test data set.

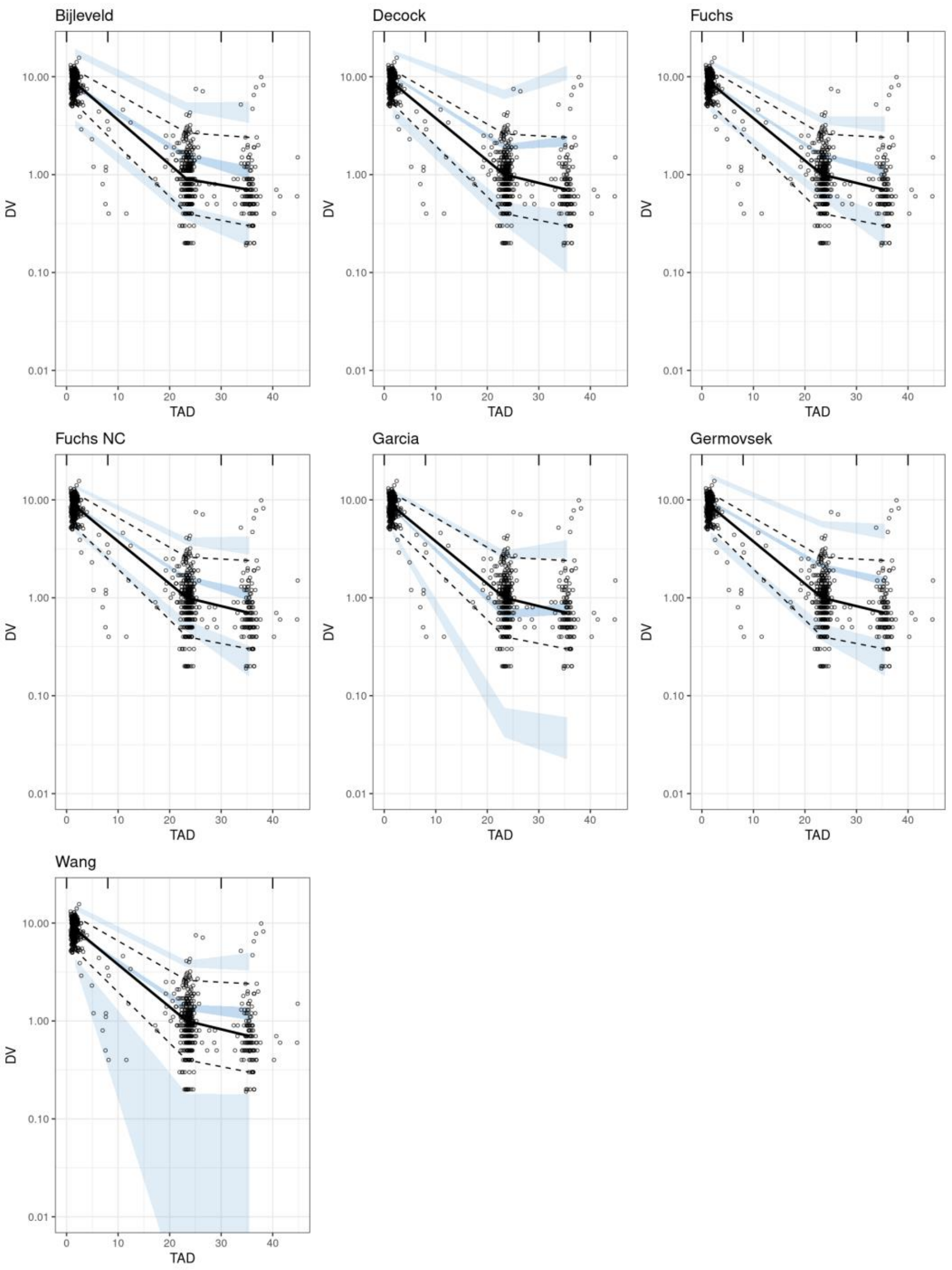

Supplemental Figure S5. Visual predictive checks (VPCs) of published models with observed gentamicin concentrations (circles) and population predictions (solid line) with corresponding 95% prediction interval (dotted lines). Semi-transparent blue fields represent model-based percentile confidence interval. BL: Bijleveld.

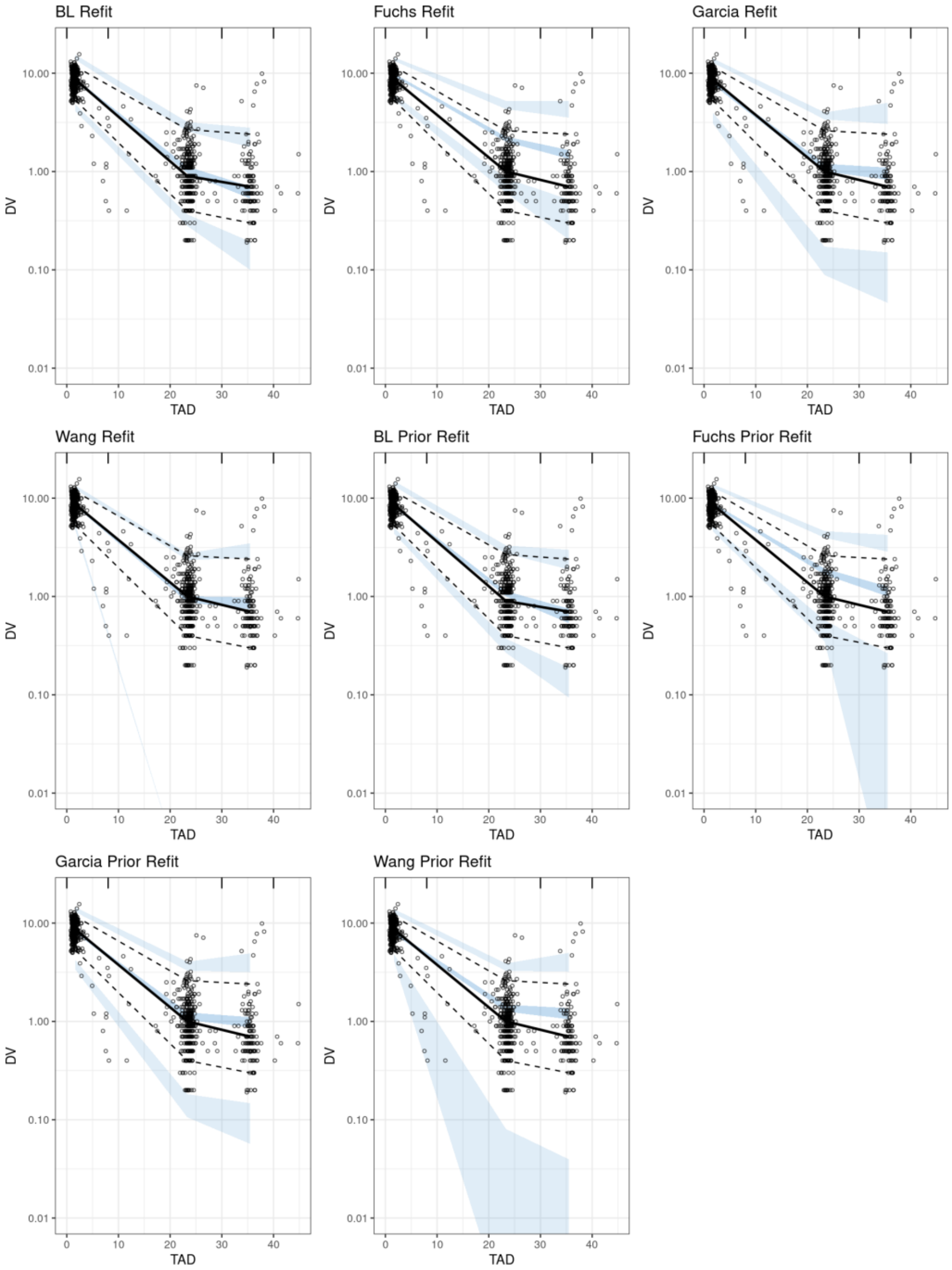

Supplemental Figure S6. Visual predictive checks (VPCs) of refitted models with observed gentamicin concentrations (circles) and population predictions (solid line) with corresponding 95% prediction interval (dotted lines). Semi-transparent blue fields represent model-based percentile confidence interval. BL: Bijleveld.

Supplemental Table S1: Model parameters from refit model with informative priors and refit model without informative priors for the Bijleveld (BL) model.

	BL published		BL with prior		BL without prior	
**Parameter**	**Estimate**	**SE**	**Estimate**	**SE**	**Estimate**	**SE**
CL	1.00	0.05	1.19	0.03	1.28	0.04
TH_PMA	1.89	0.26	1.94	0.12	1.95	0.15
V	37.4	0.04	30.93	0.87	26.37	1.47
Q	0.57	0.67	0.76	0.14	1.86	0.45
V2	17.1	0.49	13.59	2.05	21.69	3.13
add	0.18	0.21	0.06	0.01	0.06	0.01
CL_IIV	0.11	0.2	0.08	0.01	0.08	0.01
V_IIV	0.06	0.1	0.04	0.01	0.06	0

Supplemental Table S2: Model parameters from refit model with informative priors and refit model without informative priors for the Fuchs model.

	Fuchs published		Fuchs with prior		Fuchs without prior	
**Parameter**	**Estimate**	**SE**	**Estimate**	**SE**	**Estimate**	**SE**
CL	0.09	0.01	0.09	0	0.10	0.01
TH_GACL	1.87	0.03	1.91	0.05	1.83	0.17
TH_PNACL	0.05	0.06	0.05	0	0.03	0.01
V	0.91	0.02	0.87	0.02	0.92	0.02
TH_GAV1	-0.92	0.08	-0.77	0.05	-0.14	0.11
Q	0.16	0.07	0.13	0.01	0.06	0.01
V2	0.56	0.04	0.54	0.02	0.48	0.07
prop	0.03	0.24	0.03	0.01	0.03	0.01
add	0.01	0.01	0.08	0.02	0.09	0.03
CL_IIV	0.08	0.03	0.08	0.01	0.07	0.02
CL_V_IIV	0.04	0.03	0	0	0	0
V_IIV	0.03	0.01	0.03	0.01	0.01	0.01

Supplemental Table S3: Model parameters from refit model with informative priors and refit model without informative priors for the Wang model.

	**Wang published**		**Wang with prior**		**Wang without prior**	
**Parameter**	**Estimate**	**SE**	**Estimate**	**SE**	**Estimate**	**SE**
CL	4.57	0.01	4.61	0.03	5.29	0.23
V	18.1	0.01	17.8	0.15	15.35	0.79
V2	17.1	0.01	17.1	0.10	17.0	6.73
Q	1.07	0.01	1.07	0.01	0.90	0.35
add	0.18	0.01	0.32	0.03	0.33	0.11
CL_IIV	0.15	0	0.10	0.01	0.07	0.03
CL_V_IIV	0.02	0.01	0.01	0.01	0.04	0.01
V_IIV	0.24	0.01	0.15	0.01	0.05	0.01
V2_IIV	0.45	0.09	0.49	0.23	0.57	0.42
